# The efficacy and safety of lower-dose aspirin for primary and secondary prevention of cardiovascular disease in the elderly: an interim analysis of a multicenter, prospective, observational study

**DOI:** 10.3389/fcvm.2025.1615074

**Published:** 2026-01-12

**Authors:** Xiting Wang, Hong Qi, Yuan Wu, Hongliang Cong, Pida Hao, Xiqiang Liu, Yong Liu, Zhuhua Yao, Aiping Jin, Yan Hou, Nabuqi He, Yingxin Zhao, Yanmei Sun, Xuefen Qian, Keshan Liang, Huaizhong Zhang, Lili Liu, Zhengxiang Zhang, Yingwu Liu, Peng Dou, Shudong Xia, Hongwei Li, Jiuyu Yang, Jie Hu, Zhangyong Xia, Bo Liu, Hailian Jin, Xiulian Yan, Wei Miao, Huanyu Guo, Longmei Zhao, Qingtan Zhang, Tao Tian, Xibo Sun, Jianwei He, Xiaoping Chen, Zhaohui Wang, Zhenghua Zhang, Qing Liu, Jianchun Wang, Sainan Zhu, Meilin Liu

**Affiliations:** 1 Department of Geriatrics, Peking University First Hospital, Beijing, China; 2 Department of Cardiology, The Affiliated Hospital of Chifeng University, Neimenggu, China; 3 Department of Cardiology, LinYi Luozhuang Central Hospital, Linyi, Shandong, China; 4 Department of Cardiology, Tianjin Chest Hospital, Tianjin, China; 5 Department of Neurology, LinYi Luozhuang People’s Hospital, Linyi, Shandong, China; 6 Department of Cardiology, Zibo Central Hospital, Zibo, Shandong, China; 7 Department of Cardiology, Tianjin Fourth Center Hospital, Tianjin, China; 8 Department of Cardiology, Tianjin People’s Hospital, Tianjin, China; 9 Department of Geriatrics, The Second Affiliated Hospital of Xi’an Jiaotong University (Xibei Hospital), Xi'an, Shannxi, China; 10 Department of Neurology, Zhangdian People’s Hospital, Zibo, Shandong, China; 11 Department of Cardiology, Xing’anmeng Mongolian Hospital, Neimenggu, China; 12 Department of Cardiology, Beijing Anzhen Hospital Capital Medical University, Beijing, China; 13 Department of Cardiology, Chifeng Songshan Hospital, Neimenggu, China; 14 Department of Geriatrics, The First People’s Hospital of Lianyungang, Lianyungang, Jiangsu, China; 15 Department of Neurology, The People’s Hospital of Pingyi County, Linyi, Shandong, China; 16 Department of Geriatrics, Xuzhou Cancer Hospital, Xuzhou, Jiangsu, China; 17 Department of Endocrinology, Xing’anmeng People’s Hospital, Neimenggu, China; 18 Department of Cardiology, The Fourth People Hospital of Zibo, Zibo, Shandong, China; 19 Department of Cardiology, Tianjin Third Central Hospital, Tianjin, China; 20 Department of Neurology, Zibo Guangdian Hospital, Zibo, Shandong, China; 21 Department of Cardiology, The Fourth Affiliated Hospital, Zhejiang University, Yiwu, Zhejiang, China; 22 Department of Geriatrics, PKUCare Luzhong Hospital, Zibo, Shandong, China; 23 Department of Cardiology, The Second Affiliated Hospital, Inner Mongolia Minzu University, Neimenggu, China; 24 Department of Geriatrics, The Jiangxi Provincial People’s Hospital, Nanchang, Jiangxi, China; 25 Department of Neurology, Liaocheng Brian Hospital, Liaocheng, Shandong, China; 26 Department of Cardiology, Shandong Yiyangjiankang Jituan Zibo Hospital, Zibo, Shandong, China; 27 Department of Neurology, PKUCare Zibo Hospital, Zibo, Shandong, China; 28 Department of Cardiology, Zibo Gaoqing Hospital, Zibo, Shandong, China; 29 Department of Cardiology, Central Hospital Affiliated to Shandong First Medical University, Jinan, Shandong, China; 30 Department of Cardiology, Duancheng County People’s Hospital, Henan, China; 31 Department of Cardiology, Linyi Hospital of Traditional Chinese Medicine, Linyi, Shandong, China; 32 Department of Geriatrics, Binzhou Medical University Hospital, Binzhou, Shandong, China; 33 Department of Geriatrics, Linyi People’s Hospital, Linyi, Shandong, China; 34 Department of Neurology, Yidu Central Hospital of Weifang, Weifang, Shandong, China; 35 Department of Cardiology, Linyi Transportation Hospital, Linyi, Shandong, China; 36 Department of Cardiology, Taiyuan Central Hospital, Taiyuan, Shanxi, China; 37 Department of Geriatrics, Union Hospital Tongji Medical College Huazhong University of Science and Technology, Wuhan, Hubei, China; 38 Department of Occupational Disease, The Sixth People’s Hospital of Zibo, Zibo, Shandong, China; 39 Department of Cardiology, People’s Hospital of Rizhao Lanshan, Rizhao, Shandong, China; 40 Department of Geriatrics, Shandong Provincial Hospital, Jinan, Shandong, China; 41 Department of Medical Statistics, Peking University First Hospital, Beijing, China

**Keywords:** cardiovascular diseases, elderly, lower-dose aspirin, primary prevention, secondary prevention

## Abstract

**Introduction:**

Although low-dose aspirin effectively reduces atherothrombosis occurrence in individuals diagnosed with cardiovascular disease (CVD) or in those with high-risk factors, it is significantly associated with increased bleeding. No evidence has been established for a lower dose of aspirin.

**Methods:**

The Lower-dose Aspirin for Primary and Secondary Prevention of Cardiovascular Disease in the Elderly (LAPIS) is a multicenter, prospective, observational cohort study, which compared the benefits and risks in adults aged 60 years and older taking aspirin 50 or 100 mg/day for primary and secondary CVD prevention in a propensity score-matched population. The efficacy outcome was a composite of the first occurrence of major adverse cardiovascular events (MACE). The safety outcome was the first occurrence of any hemorrhagic events.

**Results:**

In this interim analysis of LAPIS, 7,021 participants were followed up for a median of 183 (95% CI 169–197) days (primary prevention cohort, 2,070; secondary prevention cohort, 4,951). After adjusting for baseline characteristics using propensity score matching, the MACE incidence did not differ significantly between the two dosage groups in either cohort. However, in the primary prevention cohort, the incidence of any bleeding [8.89 vs. 3.45 events/100 patient-years, hazard ratio (HR) 2.917, 95% confidence interval (CI) 1.719–4.952, *P* < 0.001] and gastrointestinal events (8.30 vs. 5.04 events/100 patient-years, HR 1.745, 95% CI 1.047–2.907, *P* = 0.037) was higher in the 100 mg/day group. In the secondary prevention cohort, the 100 mg/day group showed higher rates of any bleeding (9.19 vs. 6.37 events/100 patient-years, HR 1.473, 95% CI 1.087–1.998, *P* = 0.015), minor bleeding (9.10 vs. 6.06 events/100 patient-years, HR 1.541, 95% CI 1.116–2.127, *P* = 0.009), and gastrointestinal adverse events (7.10 vs. 3.53 events/100 patient-years, HR 1.943, 95% CI 1.291–2.925, *P* = 0.002).

**Conclusion:**

Aspirin 50 mg/day was associated with lower hemorrhage and gastrointestinal adverse event risks, with similar cardiovascular benefits, compared with aspirin 100 mg/day, and may be preferred to balance efficacy and safety for older Chinese adults in primary and secondary CVD prevention.

## 1 Introduction

Cardiovascular disease (CVD) is the leading cause of death in the Chinese population, causing over four million deaths annually and representing more than 40% of total mortality. The aging of the population in China exacerbates the burden of CVD, with both incidence and mortality rising dramatically. This growing demographic challenge underscores the urgent need to optimize management strategies, particularly for elderly individuals with CVD or at an elevated risk.

Aspirin, a platelet aggregation inhibitor that leads to long-lasting suppression of thromboxane A2 production by acetylated cyclooxygenase-1 (COX-1), has been recommended as a strategy for secondary prevention in the past decade owing to its demonstrated net benefit. Studies have convincingly demonstrated that low-dose aspirin (75–100 mg/day) significantly reduces the occurrence of stroke and major adverse cardiovascular events (MACE) (1–4). However, the role of low-dose aspirin in primary prevention remains debatable. In primary prevention trials assessing older adults, the benefits are often offset by the risk of hemorrhagic events (5–11). Compared with Western populations, East Asian populations using low-dose aspirin (75–100 mg/day) have an increased risk of bleeding (3, 9, 12, 13), especially in patients with severe renal impairment, as well as polypharmacy, including concomitant use of other antithrombotic or nonsteroidal anti-inflammatory drugs (NSAIDs), frailty, and other complicated comorbidities (14).

Aspirin dose is associated with bleeding risk. Therefore, optimizing the aspirin dosage to preserve its efficacy while minimizing the risk of bleeding is crucial (15, 16). It has been reported that doses of aspirin as low as 30 mg/day for a week could completely block the effects of COX-1 (17), and the effective aspirin dose ranges between 50 and 100 mg/day based on randomized controlled trials in both acute coronary syndrome and stable patients with CVD (18). Based on our previous findings (19, 20), we hypothesized that lower-dose aspirin (50 mg/day) would be non-inferior to 100 mg/day for both primary and secondary prevention of cardiovascular disease, and that it might be associated with a lower incidence of adverse events in older Chinese patients. Further studies involving larger multicenter populations are needed to confirm these hypotheses.

## 2 Material and methods

### 2.1 Study design and participants

The Lower-dose Aspirin for Primary and Secondary Prevention of Cardiovascular Disease in the Elderly (LAPIS) study (chictr.org.cn, ChiCTR1900021980) is a prospective multicenter observational cohort study performed at 80 sites covering 22 central cities in mainland China. The study was approved by the institutional ethics committee [Peking University First Hospital, approval number 2018(248)], and the rationale and design of the study have been previously published (21). Written informed consent was obtained from all patients. Patients aged 60 years and older who required long-term aspirin therapy for primary or secondary prevention based on clinical assessment were included. The exclusion criteria were as follows: (1) history of aspirin-sensitive asthma or allergies to aspirin, salicylic acid, or NSAIDs; (2) life expectancy of ≤3 years. The study was conducted in accordance with the principles of the Declaration of Helsinki.

### 2.2 Variable definition and data collection

Data at baseline enrollment and follow-up were systematically collected into electronic case report forms using an electronic data capture system according to standard procedures. All essential information was recorded in detail and managed by independent clinical research coordinators of the Shanghai Ashermed Healthcare Communications Ltd. Demographic information included age, sex, smoking history, and alcohol intake. Medical history included previous history of coronary heart disease, nonfatal myocardial infarction (MI), unstable angina, vascular diseases requiring surgical/interventional revascularization, nonfatal stroke, transient ischemic attack (TIA), hemorrhagic events, gastrointestinal disease, and comorbidities (hypertension, diabetes mellitus, or dyslipidemia). Laboratory indication included estimated glomerular filtration rate (eGFR), glycosylated hemoglobin (HbA1C), serum lipids (triglycerides, total cholesterol, low-density lipoprotein cholesterol, and high-density lipoprotein cholesterol), and high-sensitivity C reactive protein (hsCRP). Prescription information included the status of aspirin intake, concomitant use of other antiplatelets and anticoagulants, β-blockers, statins, angiotensin-converting enzyme inhibitors (ACEIs), angiotensin receptor blockers (ARBs), aldosterone receptor antagonists (MRAs), proton pump inhibitors (PPIs), and histamine 2 receptor antagonists (H2RAs). Patients without a history of coronary heart disease, stroke, transient ischemic attack, or peripheral artery disease were defined as taking aspirin for primary prevention. Hemorrhage history was defined as bleeding at any site and the severity of bleeding events. The timing of prior percutaneous coronary intervention (PCI) was not collected in the electronic case report forms, therefore, patients with recent PCI were not specifically identified.

### 2.3 Follow-up and outcomes

The LAPIS procedures did not interfere with the aspirin therapy strategy administered to the patients. Physicians determined the aspirin dosage and treatment duration based on the patient's clinical condition. Follow-ups were conducted by physicians during face-to-face visits or through telephone calls and online contact. Routine follow-ups were performed at the 1st month and 3rd month and every 6 months thereafter until the end of the LAPIS (at least nine times). An independent group of physicians examined and verified all events at each visit. The efficacy outcome was a composite of the first occurrence of MACE, including unstable angina, nonfatal myocardial infarction, nonfatal stroke, arteriosclerotic diseases requiring surgery or intervention, cardiovascular death (excluding intracranial hemorrhage), and TIA. The safety outcome was the first occurrence of hemorrhagic events, defined as a composite of fatal bleeding (Bleeding Academic Research Consortium, BARC, type 5), major bleeding (BARC, type 3–4), and minor bleeding (BARC, type 1–2) (22). In addition, data on adverse gastrointestinal events associated with aspirin, including new-onset gastroduodenal ulcer, reflux esophagitis, erosive gastritis, stomach or abdominal discomfort, pain, pressure, heartburn, and nausea, were collected for safety analyses. The time to the event was defined as the number of days from the date of enrollment to the date of confirmation of the event. Participants who did not experience the event of interest were censored at the time of loss to follow-up or at the end of the study period. For analyses in which cardiovascular death was the event of interest, cardiovascular death was treated as the event, whereas deaths from other causes were treated as censoring events.

### 2.4 Statistical analysis

Statistical analyses were performed using IBM SPSS Statistics software (version 27.0; IBM, Armonk, NY, USA). All analyses were conducted on populations taking aspirin for primary or secondary prevention. Continuous variables at baseline are presented as the mean ± standard deviation (SD) or median (interquartile range) and were compared using an independent samples *t*-test or the Mann‒Whitney *U*-test, whereas categorical data are presented as frequencies and percentages and were compared using Pearson's *χ*^2^-test or Fisher's exact test when expected counts were <5 in any cell. To reduce potential confounding between the aspirin 50 and 100 mg/day groups, propensity score matching (PSM) was performed at a 1:1 ratio using propensity scores estimated from multivariable logistic regression, with aspirin dose as the dependent variable. Covariates included in the propensity score models were selected *a priori* based on clinical relevance and their potential to confound the association between aspirin dose and outcomes. Separate propensity score models were constructed for the primary and secondary prevention cohorts. For the primary prevention cohort, covariates included age, sex, current smoking status, current alcohol consumption, diabetes mellitus, concomitant use of other antiplatelet agents, ACEIs, ARBs, MRAs, and statins, as well as laboratory parameters including total cholesterol (TCHO), low-density lipoprotein cholesterol (LDL-C), and eGFR. For the secondary prevention cohort, covariates included age, sex, current smoking status, current alcohol consumption, medical history [coronary heart disease, myocardial infarction, unstable angina pectoris, PCI or coronary artery bypass grafting (CABG), stroke, hypertension, diabetes mellitus, and history of hemorrhage], concomitant use of other antiplatelet agents, anticoagulants, PPI/H2RA, and statins, as well as laboratory parameters including eGFR, TCHO, LDL-C, and HbA1c. After matching, baseline characteristics between the two aspirin dose groups were compared to assess covariate balance, and all subsequent analyses were conducted in the matched cohorts. Median follow-up time was estimated using the reverse Kaplan–Meier method. Events were presented as both raw incidence proportions (patients with events/number of treated patients) and incidence rates (patients with events per 100 patient-years). Time-to-event data were depicted using Kaplan–Meier survival curves, and the differences between the curves were assessed using the log-rank test. Multivariable Cox proportional hazards models were developed to identify the independent predictors of bleeding events after adjusting for variables known to be strongly associated with the risk of bleeding events or differed significantly on univariable analysis. Hazard ratios (HRs) and 95% confidence intervals (CIs) were calculated. Statistical significance was defined for all analyses as a two-sided *P*-value of less than 0.05.

## 3 Results

### 3.1 Participants and follow-up

**Figure 1:**
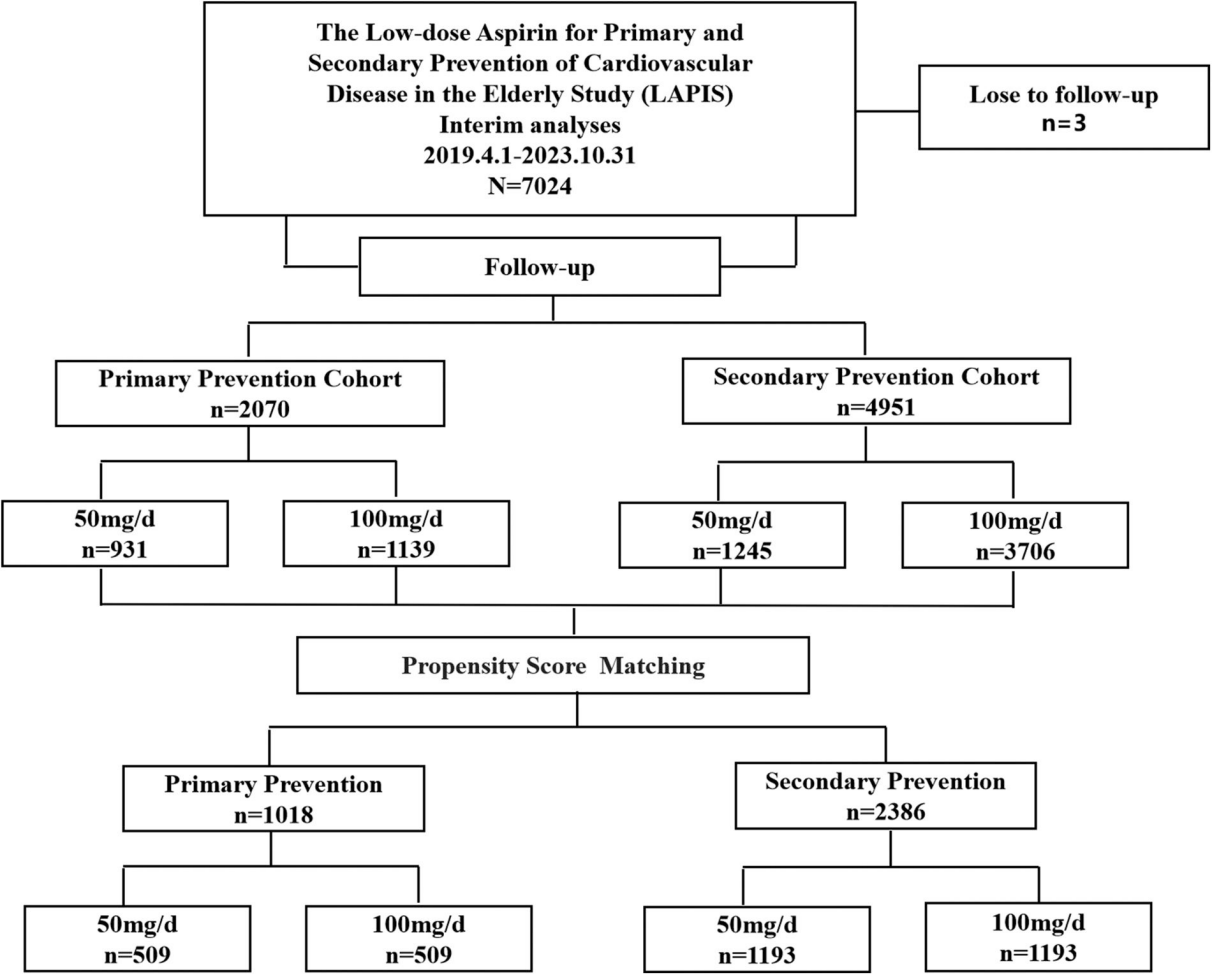
Flow diagram.

After excluding patients lost to follow-up, 7,021 participants were enrolled in the LAPIS, with a median follow-up of 183 (95% CI 169–197) days, from April 1, 2019, to October 31, 2023. In total, 2,070 and 4,951 participants were included in the primary and secondary prevention cohorts, respectively (Figure 1). Of these, 2,176 patients (30.99%) received aspirin 50 mg/day, and 4,845 (69.01%) patients received aspirin 100 mg/day. New-onset MACE occurred in 145 patients (2.07%), bleeding events occurred in 554 patients (7.89%), and gastrointestinal adverse events occurred in 468 patients (6.67%). There were 96 deaths (1.37%).

### 3.2 Primary prevention

#### 3.2.1 Baseline characteristics

After propensity score matching, the aspirin 50 and 100 mg/day groups were well balanced with respect to baseline demographic and clinical characteristics. No statistically significant differences in baseline variables were observed between the two groups after matching. The demographic and clinical characteristics of the participants at baseline are presented in Table 1.

**Table 1:** Baseline characteristics for 50 and 100 mg/day aspirin in primary prevention.

Characteristic	Before PSM adjustment			After PSM adjustment		
	Aspirin 50 mg/day	Aspirin 100 mg/day	*P*	Aspirin 50 mg/day	Aspirin 100 mg/day	*P*
Number of patients	931	1,139		509	509	
Demographics						
Age, years	68.59 ± 6.74	69.38 ± 6.51	0.007*	69.11 ± 7.14	69.41 ± 6.70	0.491
≥75, *n* (%)	173 (18.6%)	247 (21.7%)	0.081	107 (21.0%)	113 (22.2%)	0.648
Male, *n* (%)	424 (45.50%)	571 (50.1%)	0.038*	253 (49.7%)	261 (51.3%)	0.616
Current smoking, *n* (%)	97 (10.4%)	238 (20.9%)	<0.001*	66 (13.0%)	65 (12.8%)	0.925
Current drinking, *n* (%)	113 (12.1%)	295 (25.9%)	<0.001*	82 (16.1%)	85 (16.7%)	0.800
Medical history						
Hypertension, *n* (%)	596 (64.0%)	733 (64.4%)	0.873	315 (61.9%)	337 (66.2%)	0.151
Diabetes mellitus, *n* (%)	196 (21.1%)	312 (27.4%)	<0.001*	115 (22.6%)	115 (22.6%)	1.000
Dyslipidemia, *n* (%)	131 (14.1%)	162 (14.2%)	0.921	70 (13.8%)	57 (11.2%)	0.218
Gastrointestinal disease, *n* (%)	75 (8.1%)	94 (8.3%)	0.871	49 (9.6%)	46 (9.0%)	0.747
Hemorrhage history, *n* (%)	10 (1.1%)	16 (1.4%)	0.502	7 (1.4%)	4 (0.8%)	0.547
Concomitant medication						
Concomitant use of other antiplatelets, *n* (%)	26 (2.8%)	163 (14.3%)	<0.001*	21 (4.1%)	19 (3.7%)	0.747
Clopidogrel, *n* (%)	25 (2.7%)	157 (13.8%)	<0.001*	21 (4.1%)	19 (3.7%)	0.747
Ticagrelor, *n* (%)	0	4 (0.4%)	0.132	0	0	-
Cilostazol, *n* (%)	1 (0.1%)	2 (0.2%)	1.000	0	0	-
Concomitant use of anticoagulants, *n* (%)	9 (1.0%)	6 (0.5%)	0.240	2 (0.4%)	2 (0.4%)	1.000
Rivaroxaban, *n* (%)	4 (0.4%)	1 (0.1%)	0.181	2 (0.4%)	0	0.500
Dabigatran, *n* (%)	1 (0.1%)	0	0.450	0	0	-
Warfarin *n* (%)	1 (0.1%)	1 (0.1%)	1.000	0	0	-
Low molecular weight heparin, *n* (%)	3 (0.3%)	4 (0.4%)	1.000	0	2 (0.4%)	0.500
β-blocker, *n* (%)	124 (13.3%)	140 (12.3%)	0.486	77 (15.1%)	72 (14.1%)	0.658
ACEI/ARB/MRA, *n* (%)	107 (11.5%)	214 (18.8%)	<0.001*	84 (16.5%)	80 (15.7%)	0.733
Statin, *n* (%)	518 (55.6%)	776 (68.1%)	<0.001*	340 (66.8%)	322 (63.3%)	0.237
PPI/H2RA, *n* (%)	76 (8.2%)	69 (6.1%)	0.062	55 (10.8%)	42 (8.3%)	0.165
Laboratory parameters						
eGFR (mL/min/1.73 m^2^)	95.81 ± 24.70	98.57 ± 33.34	0.046*	95.69 ± 24.34	97.91 ± 26.67	0.179
TG (mmol/L)	1.53 ± 0.93	1.59 ± 1.03	0.247	1.44 ± 0.86	1.46 ± 0.92	0.747
TCHO (mmol/L)	4.76 ± 1.17	4.58 ± 1.17	0.001*	4.22 ± 0.82	4.21 ± 0.85	0.886
LDL-C (mmol/L)	2.45 ± 0.59	2.29 ± 0.63	<0.001*	2.38 ± 0.60	2.36 ± 0.63	0.506
HDL-C (mmol/L)	1.24 ± 0.33	1.24 ± 0.31	0.721	1.21 ± 0.33	1.21 ± 0.30	0.856
HbA1c (%)	6.10 (5.70, 7.00)	6.20 (5.70, 7.11)	0.403	6.00 (5.70, 6.80)	6.10 (5.60, 7.00)	0.659
hsCRP (mg/L)	1.17 (0.50, 3.75)	1.20 (0.50, 2.70)	0.508	0.97 (0.50, 3.60)	1.15 (0.50, 2.35)	0.777

Data are presented as mean ± standard deviation or median (interquartile range) for continuous variables and percentage (%) for categorical variables. ACEI, angiotensin-converting enzyme inhibitors; ARB, angiotensin receptor blockers; eGFR, estimated glomerular filtration rate; H2RA, histamine 2 receptor antagonist; HbA1C, glycosylated hemoglobin; HDL-C, high density lipoprotein cholesterol; hsCRP, high sensitivity C reactive protein; LDL-C, low density lipoprotein cholesterol; MRA, aldosterone receptor antagonist; PPI, proton pump inhibitors; PSM, propensity score matching; TCHO, total cholesterol; TG, triglyceride.

* *P*-value <0.05 was considered nominally statistically significant.

#### 3.2.2 Efficacy and safety outcomes

**Figure 2:**
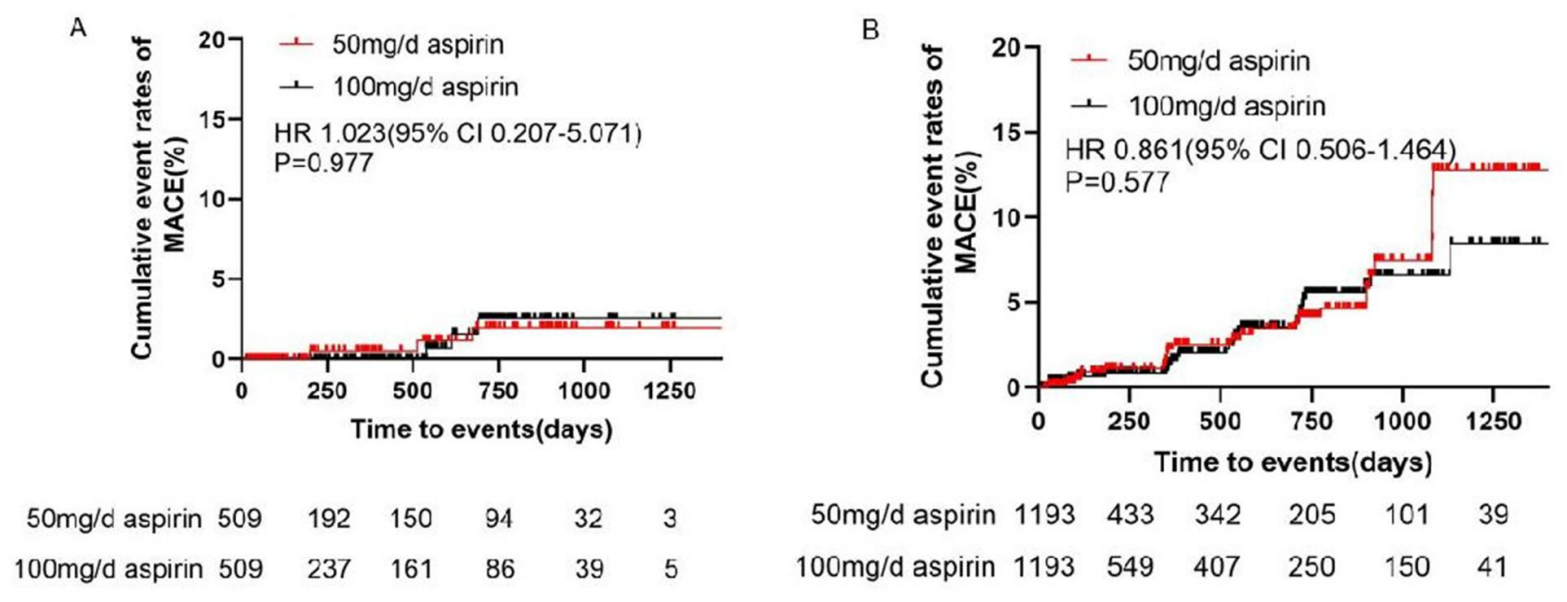
Cumulative event rates of MACE in **(A)** primary prevention and **(B)** secondary prevention. CI, confidence interval; HR, hazard ratio; MACE, major cardiovascular events.

**Figure 3:**
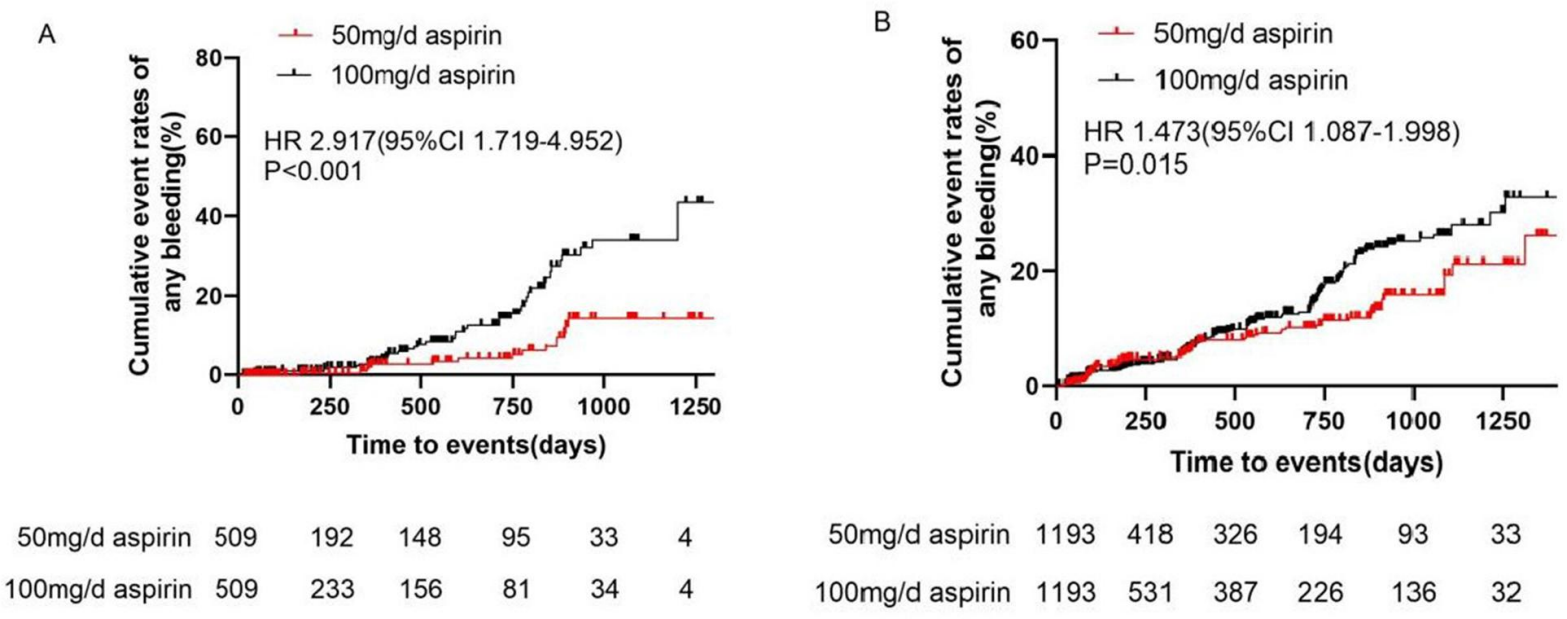
Cumulative event rates of bleeding events in **(A)** primary prevention and **(B)** secondary prevention. CI, confidence interval; HR, hazard ratio.

After adjustment, the incidence of MACE did not differ significantly between the 100 and 50 mg/day groups (0.65 vs. 0.70 events/100 patient-years, HR 1.023, 95% CI 0.207–5.071, *P* = 0.977) (Table 2 and Figure 2). Bleeding events occurred in 18 (1.93%) patients in the 50 mg group and 107 (9.39%) patients in the 100 mg group. After adjustment, the incidence rates of any bleeding events, minor bleeding events (8.89 vs. 3.45 events/100 patient-years, HR 2.917, 95% CI 1.719–4.952, *P* < 0.001), and gastrointestinal adverse events (8.30 vs. 5.04 events/100 patient-years, HR 1.745, 95% CI 1.047–2.907, *P* = 0.037) were higher in the 100 mg group than in the 50 mg group (Table 2 and Figure 3).

**Table 2:** Efficacy and safety outcomes for 50 and 100 mg/day aspirin in primary prevention.

Outcome	Before PSM adjustment				After PSM adjustment			
	No. of patients with event/No. of treated patients (Incidence rate, events/100 patient-years)		HR (95% CI) 100 vs. 50 mg/day	*P*	No. of patients with event/No. of treated patients (Incidence rate, events/100 patient-years)		HR (95% CI) 100 vs. 50 mg/day	*P*
	Aspirin 50 mg/day (*n* = 931)	Aspirin 100 mg/day (*n* = 1,139)			Aspirin 50 mg/day (*n* = 509)	Aspirin 100 mg/day (*n* = 509)		
Efficacy outcomes								
MACE	5/931 (0.63)	9/1,139 (0.82)	1.197 (0.390–3.671)	0.753	3/509 (0.70)	3/509 (0.65)	1.023 (0.207–5.071)	0.977
UA	1/931 (0.13)	2/1,139 (0.18)	0.717 (0.045–11.471)	0.814	1/509 (0.23)	1/509 (0.02)	0.894 (0.056–14.333)	0.937
Nonfatal MI	0	0	-	-	0	0	-	-
Nonfatal stroke	2/931 (0.25)	2/1,139 (0.18)	0.715 (0.099–5.171)	0.740	2/509 (0.46)	1/509 (0.02)	0.536 (0.048–5.926)	0.611
Arteriosclerotic diseases requiring surgery or intervention	2/931 (0.25)	1/1,139 (0.09)	0.393 (0.036–4.336)	0.446	0	0	-	-
Cardiovascular death	0	3/1,139 (0.27)	-	-	0	1/509 (0.02)	-	-
TIA	0	1/1,139 (0.09)	-	-	0	0	-	-
Safety outcomes								
Any bleeding	18/931 (2.27)	107/1,139 (9.90)	4.487 (2.721–7.400)	<0.001*	15/509 (3.45)	40/509 (8.89)	2.917 (1.719–4.952)	<0.001*
Minor bleeding (BARC 1–2)	18/931 (2.27)	106/1,139 (9.81)	4.445 (2.695–7.333)	<0.001*	15/509 (3.45)	40/509 (8.89)	2.917 (1.719–4.952)	<0.001*
Major bleeding (BARC 3–4)	0	1/1,139 (0.09)	-	-	0	0	-	-
Fatal bleeding (BARC 5)	0	0	-	-	0	0	-	-
Gastrointestinal adverse events	26/931 (3.35)	125/1,139 (11.61)	3.483 (2.282–5.318)	<0.001*	21/509 (5.04)	38/509 (8.30)	1.745 (1.047–2.907)	0.037*

BARC, Bleeding Academic Research Consortium; CI, confidence interval; HR, hazard ratio; MACE, major cardiovascular events; MI, myocardial infarction; PSM, propensity score matching; TIA, transient ischemic attacks; UA, unstable angina pectoris.

* *P*-value <0.05 was considered nominally statistically significant.

### 3.3 Secondary prevention

#### 3.3.1 Baseline characteristics

After propensity score matching, the aspirin 50 and 100 mg/day groups were well balanced with respect to baseline demographic and clinical characteristics. No statistically significant differences in baseline variables were observed between the two groups after matching. The demographic and clinical characteristics of the participants at baseline are shown in Table 3.

**Table 3:** Baseline characteristics for 50 and 100 mg/day aspirin in secondary prevention.

Characteristic	Before PSM adjustment			After PSM adjustment		
	Aspirin 50 mg/day	Aspirin 100 mg/day	*P*	Aspirin 50 mg/day	Aspirin 100 mg/day	*P*
Number of patients	1,245	3,706		1,193	1,193	
Demographics						
Age, years	71.27 ± 8.13	70.26 ± 6.98	<0.001*	71.15 ± 8.06	71.17 ± 7.41	0.962
≥75, *n* (%)	386 (31.0%)	970 (26.2%)	<0.001*	362 (30.3%)	371 (31.1%)	0.690
Male, *n* (%)	571 (45.9%)	2,152 (58.1%)	<0.001*	549 (46.0%)	574 (48.1%)	0.305
Current smoking, *n* (%)	134 (10.8%)	776 (20.9%)	<0.001*	132 (11.1%)	137 (11.5%)	0.746
Current drinking, *n* (%)	121 (9.7%)	788 (21.3%)	<0.001*	121 (10.1%)	130 (10.9%)	0.548
Medical history						
Coronary heart disease, *n* (%)	1,102 (88.5%)	3,092 (83.4%)	<0.001*	1,050 (88.0%)	1,045 (87.6%)	0.754
MI history, *n* (%)	101 (8.1%)	668 (18.0%)	<0.001*	101 (8.5%)	98 (8.2%)	0.824
UA, *n* (%)	323 (25.9%)	1,434 (38.7%)	<0.001*	322 (27.0%)	325 (27.2%)	0.890
PCI/CABG history, *n* (%)	156 (12.5%)	1,027 (27.7%)	<0.001*	155 (13.0%)	154 (12.9%)	0.951
Stroke history, *n* (%)	223 (17.9%)	1,030 (27.8%)	<0.001*	222 (18.6%)	227 (19.0%)	0.793
TIA history, *n* (%)	55 (4.4%)	140 (3.8%)	0.315	54 (4.5%)	64 (5.4%)	0.345
Hypertension, *n* (%)	849 (68.2%)	2,700 (72.9%)	0.002*	822 (68.9%)	802 (67.2%)	0.380
Diabetes mellitus, *n* (%)	327 (26.3%)	1,258 (33.9%)	<0.001*	319 (26.7%)	320 (26.8%)	0.963
Dyslipidemia, *n* (%)	291 (23.4%)	910 (24.6%)	0.400	284 (23.8%)	269 (22.5%)	0.467
Gastrointestinal disease, *n* (%)	220 (17.7%)	600 (16.2%)	0.224	211 (17.7%)	213 (17.9%)	0.915
Hemorrhage history, *n* (%)	73 (5.9%)	117 (3.2%)	<0.001*	67 (5.6%)	61 (5.1%)	0.586
Concomitant medication						
Concomitant use of other antiplatelets, *n* (%)	216 (17.3%)	1,673 (45.1%)	<0.001*	216 (18.1%)	245 (20.5%)	0.133
Clopidogrel, *n* (%)	170 (12.7%)	1,227 (33.1%)	<0.001*	170 (14.2%)	186 (15.6%)	0.358
Ticagrelor, *n* (%)	44 (3.5%)	442 (11.9%)	<0.001*	44 (3.7%)	58 (4.9%)	0.157
Cilostazol, *n* (%)	2 (0.2%)	4 (0.1%)	1.000	2 (0.2%)	1 (0.1%)	1.000
Concomitant use of anticoagulants, *n* (%)	62 (5.0%)	79 (2.1%)	<0.001*	52 (4.4%)	50 (4.2%)	0.840
Rivaroxaban, *n* (%)	40 (3.2%)	47 (1.3%)	<0.001*	33 (2.8%)	27 (2.3%)	0.433
Dabigatran, *n* (%)	8 (0.6%)	3 (0.1%)	0.001*	8 (0.7%)	2 (0.2%)	0.109
Warfarin *n* (%)	3 (0.2%)	6 (0.2%)	0.700	2 (0.2%)	5 (0.4%)	0.452
Low molecular weight heparin, *n* (%)	11 (0.9%)	23 (0.6%)	0.427	9 (0.8%)	16 (1.3%)	0.159
β-blocker, *n* (%)	501 (40.2%)	1,567 (42.3%)	0.206	488 (40.9%)	442 (37.0%)	0.053
ACEI/ARB/MRA, *n* (%)	352 (28.3%)	1,145 (30.9%)	0.081	332 (27.8%)	318 (26.7%)	0.520
Statin, *n* (%)	952 (76.5%)	3,202 (86.4%)	<0.001*	937 (78.5%)	916 (76.8%)	0.302
PPI/H2RA, *n* (%)	337 (27.1%)	1,167 (31.5%)	0.003*	322 (27.0%)	334 (28.0%)	0.582
Laboratory parameters						
eGFR (mL/min/1.73 m^2^)	88.23 ± 23.50	91.02 ± 21.50	<0.001*	88.36 ± 23.09	90.26 ± 21.82	0.051
TG (mmol/L)	1.56 ± 1.14	1.55 ± 1.10	0.801	1.56 ± 1.15	1.54 ± 1.05	0.666
TCHO (mmol/L)	4.35 ± 1.14	4.19 ± 1.15	<0.001*	4.34 ± 1.15	4.29 ± 1.19	0.316
LDL-C (mmol/L)	2.29 ± 0.63	2.17 ± 0.65	<0.001*	2.27 ± 0.63	2.21 ± 0.66	0.066
HDL-C (mmol/L)	1.17 ± 0.31	1.17 ± 0.31	0.513	1.18 ± 0.31	1.20 ± 0.31	0.058
HbA1c (%)	6.10 (5.70, 7.00)	6.30 (5.80, 7.30)	0.022*	6.10 (5.70, 7.00)	6.10 (5.70, 7.00)	0.890
hsCRP (mg/L)	1.35 (0.63, 3.34)	1.20 (0.51, 3.30)	0.139	1.32 (0.63, 3.33)	1.19 (0.50, 3.02)	0.075

Data are presented as mean ± standard deviation or median (interquartile range) for continuous variables and percentage (%) for categorical variables. ACEI, angiotensin-converting enzyme inhibitors; ARB, angiotensin receptor blockers; CABG, coronary artery bypass grafting; eGFR, estimated glomerular filtration rate; H2RA, histamine 2 receptor antagonist; HbA1C, glycosylated hemoglobin; HDL-C, high density lipoprotein cholesterol; hsCRP, high sensitivity C reactive protein; LDL-C, low density lipoprotein cholesterol; MI, myocardial infarction; MRA, aldosterone receptor antagonist; PCI, percutaneous coronary intervention; PPI, proton pump inhibitors; PSM, propensity score matching; TCHO, total cholesterol; TG, triglyceride; TIA, transient ischemic attacks; UA, unstable angina pectoris.

* *P*-value <0.05 was considered nominally statistically significant.

#### 3.3.2 Efficacy and safety outcomes

After adjustment, the incidence of MACE did not differ significantly between the 100 and 50 mg/day groups (2.31 vs. 2.71 events/100 patient-years, HR 0.861, 95% CI 0.506–1.464, *P* = 0.577). Additionally, there were no differences in the incidences of unstable angina pectoris, nonfatal myocardial infarction, nonfatal stroke, arteriosclerotic diseases requiring surgery or intervention, cardiovascular death, or TIA between the two groups (Table 4 and Figure 2). Bleeding events occurred in 63 (5.06%) patients in the 50 mg group and 366 (9.88%) patients in the 100 mg group. After adjustment, the incidence rates of any bleeding events (9.19 vs. 6.37 events/100 patient-years, HR 1.473, 95% CI 1.087–1.998, *P* = 0.015), minor bleeding events (9.10 vs. 6.06 events/100 patient-years, HR 1.541, 95% CI 1.116–2.127, *P* = 0.009), and gastrointestinal adverse events (7.10 vs. 3.53 events/100 patient-years, HR 1.943, 95% CI 1.291–2.925, *P* = 0.002) were higher in the 100 mg group than in the 50 mg group (Table 4 and Figure 3).

**Table 4:** Efficacy and safety outcomes for 50 and 100 mg/day aspirin in secondary prevention.

Outcome	Before PSM adjustment				After PSM adjustment			
	No. of patients with event/No. of treated patients (Incidence rate, events/100 patient-years)		HR (95% CI) 100 vs. 50 mg/day	*P*	No. of patients with event/No. of treated patients (Incidence rate, events/100 patient-years)		HR (95% CI) 100 vs. 50 mg/day	*P*
	Aspirin 50 mg/day (*n* = 1,245)	Aspirin 100 mg/day (*n* = 3,706)			Aspirin 50 mg/day (*n* = 1,193)	Aspirin 100 mg/day (*n* = 1,193)		
Efficacy outcomes								
MACE	28/1,245 (2.75)	103/3,706 (2.73)	1.017 (0.669–1.545)	0.939	27/1,193 (2.71)	28/1,193 (2.31)	0.861 (0.506–1.464)	0.577
UA	12/1,245 (1.18)	44/3,706 (1.17)	1.008 (0.532–1.910)	0.980	12/1,193 (1.20)	12/1,193 (0.99)	0.800 (0.359–1.781)	0.584
Nonfatal MI	4/1,245 (0.39)	10/3,706 (0.27)	0.680 (0.213–2.170)	0.515	4/1,193 (0.40)	3/1,193 (0.25)	0.637 (0.143–2.848)	0.555
Nonfatal stroke	5/1,245 (0.49)	25/3,706 (0.66)	1.347 (0.515–3.519)	0.544	4/1,193 (0.40)	5/1,193 (0.41)	1.036 (0.278–3.862)	0.957
Arteriosclerotic diseases requiring surgery or intervention	3/1,245 (0.29)	12/3,706 (0.32)	1.137 (0.320–4.036)	0.842	3/1,193 (0.30)	3/1,193 (0.25)	0.879 (0.177–4.359)	0.875
Cardiovascular death	2/1,245 (0.20)	9/3,706 (0.24)	1.174 (0.253–5.435)	0.838	2/1,193 (0.20)	3/1,193 (0.25)	1.197 (0.200–7.175)	0.844
TIA	2/1,245 (0.20)	3/3,706 (0.08)	0.520 (0.081–3.323)	0.489	2/1,193 (0.20)	2/1,193 (0.17)	0.959 (0.129–7.103)	0.967
Safety outcomes								
Any bleeding	63/1,245 (6.45)	366/3,706 (10.12)	1.598 (1.221–2.092)	<0.001*	61/1,193 (6.37)	106/1,193 (9.19)	1.473 (1.087–1.998)	0.015*
Minor bleeding (BARC 1–2)	60/1,245 (6.15)	363/3,706 (10.04)	1.664 (1.263–2.191)	<0.001*	58/1,193 (6.06)	105/1,193 (9.10)	1.541 (1.116–2.127)	0.009*
Major bleeding (BARC 3–4)	3/1,245 (0.31)	2/3,706 (0.05)	0.194 (0.032–1.163)	0.073	3/1,193 (0.31)	0	-	-
Fatal bleeding (BARC 5)	0	1/3,706 (0.03)	-	-	0	1/1,193 (0.09)	-	-
Gastrointestinal adverse events	41/1,245 (3.65)	276/3,706 (8.37)	2.386 (1.713–3.325)	<0.001*	39/1,193 (3.53)	76/1,193 (7.10)	1.943 (1.291–2.925)	0.002*

BARC, Bleeding Academic Research Consortium; CI, confidence interval; HR, hazard ratio; MACE, major cardiovascular events; MI, myocardial infarction; PSM, propensity score matching; TIA, transient ischemic attacks; UA, unstable angina pectoris.

* *P*-value <0.05 was considered nominally statistically significant.

### 3.4 Analyses of risk factors for hemorrhagic events

The results from the univariable Cox regression analysis were as follows: aspirin dose, secondary prevention, current smoking, alcohol consumption, history of dyslipidemia, gastrointestinal diseases, and hemorrhage, and concomitant use of other antiplatelets, anticoagulants, β-blockers, ACEI/ARB/MRA, statins, and PPI/H2RA were associated with increased bleeding risk. In the multivariable Cox regression analysis, we found that aspirin dose (100 vs. 50 mg/day, HR 1.714, 95% CI 1.214–2.422, *P* = 0.002), history of dyslipidemia (HR 2.151; 95% CI 1.626–2.846, *P* < 0.001) and hemorrhage (HR 1.605; 95% CI 1.075–2.397, *P* = 0.021), and concomitant use of other antiplatelet (HR 1.613, 95% CI 1.202–2.165, *P* = 0.001) and anticoagulant drugs (HR 2.310, 95% CI 1.407–3.792, *P* < 0.001) were independent risk factors for bleeding (Table 5).

**Table 5:** Features associated with bleeding events by univariable and multivariable Cox analysis.

Characteristic	Univariable Cox analysis		Multivariable Cox analysis	
	HR (95% CI)	*P*	HR (95% CI)	*P*
Sex (male vs. female)	0.874 (0.737–1.035)	0.119		
Age (years)	0.287 (0.995–1.018)	1.006		
Aspirin dose (100 vs. 50 mg/day)	2.303 (1.738–2.793)	<0.001*	1.714 (1.214–2.422)	0.002*
Prevention level (secondary vs. primary)	1.327 (1.086–1.621)	0.006*	0.818 (0.566–1.182)	0.285
Diabetes mellitus (yes vs. no)	0.845 (0.702–1.015)	0.072		
Hypertension (yes vs. no)	1.707 (0.868–3.355)	0.121		
Dyslipidemia (yes vs. no)	1.655 (1.387–1.974)	<0.001*	2.151 (1.626–2.846)	<0.001*
Gastrointestinal disease (yes vs. no)	1.542 (1.273–1.868)	<0.001*	1.107 (0.826–1.483)	0.497
Hemorrhage history (yes vs. no)	2.236 (1.697–2.945)	<0.001*	1.605 (1.075–2.397)	0.021*
Current smoking (yes vs. no)	3.002 (2.530–3.561)	<0.001*	1.341 (0.961–1.871)	0.085
Current drinking (yes vs. no)	2.742 (2.319–3.242)	<0.001*	1.261 (0.936–1.698)	0.128
β-blocker (yes vs. no)	1.338 (1.127–1.588)	<0.001*	1.243 (0.940–1.645)	0.127
ACEI/ARB/MRA (yes vs. no)	1.233 (1.026–1.481)	0.025*	1.147 (0.883–1.490)	0.305
Statin (yes vs. no)	1.906 (1.487–2.443)	<0.001*	0.757 (0.530–1.083)	0.128
PPI/H2RA (yes vs. no)	1.311 (1.084–1.586)	0.005*	1.240 (0.925–1.663)	0.150
Concomitant use of other antiplatelets (yes vs. no)	1.570 (1.320–1.867)	<0.001*	1.613 (1.202–2.165)	0.001*
Concomitant use of anticoagulants (yes vs. no)	2.267 (1.537–3.344)	<0.001*	2.310 (1.407–3.792)	<0.001*
TG (mmol/L)	0.984 (0.902–1.074)	0.722		
TCHO (mmol/L)	0.965 (0.894–1.041)	0.354		
LDL-C (mmol/L)	0.888 (0.769–1.025)	0.105		
HDL-C (mmol/L)	0.882 (0.664–1.172)	0.386		
eGFR (mL/min.1.73 m^2^)	0.999 (0.996–1.003)	0.610		
HbA1C (>6% vs. ≤6%)	0.837 (0.654–1.071)	0.158		
hsCRP (mg/L)	1.001 (0.991–1.011)	0.823		

ACEI, angiotensin-converting enzyme inhibitors; ARB, angiotensin receptor blockers; CI, confidence interval; eGFR, estimated glomerular filtration rate; H2RA, histamine 2 receptor antagonist; HbA1C, glycosylated hemoglobin; HDL-C, high density lipoprotein cholesterol; HR, hazard ratio; hsCRP, high sensitivity C reactive protein; LDL-C, low density lipoprotein cholesterol; MRA, aldosterone receptor antagonist; PPI, proton pump inhibitors; TCHO, total cholesterol; TG, triglyceride.

* *P*-value <0.05 was considered nominally statistically significant.

## 4 Discussion

To the best of our knowledge, the LAPIS is the first nationwide, large-scale, multicenter, prospective observational cohort study to evaluate the efficacy and safety of different doses of aspirin for the primary and secondary prevention of CVD in older Chinese individuals. In this interim analysis of LAPIS, we found that aspirin 50 mg/day leads to similar cardiovascular benefits but fewer bleeding events than aspirin 100 mg/day in Chinese individuals aged 60 years and older.

In clinical applications, an increasing number of physicians tend to prescribe aspirin at lower dosages than recommended because of concerns about bleeding. The obtained data reflected the real-world decision-making tendency of clinicians to use aspirin for antiplatelet therapy in older adults. Lower-dose aspirin (50 mg/day) is used more frequently for older patients at a high risk of bleeding to balance the efficacy and safety of antithrombotic therapy, including for patients aged 75 years and older, women, patients with a history of bleeding, and those who require concomitant use of anticoagulant therapy. As stated above, the lowest effective dose of aspirin for long-term antiplatelet prophylaxis ranges between 50 and 100 mg/day, consistent with the saturability of platelet COX-1 inactivation at low doses (17), which was based on indirect comparisons of randomized controlled trials employing different aspirin dosing regimens (23), as well as on head-to-head randomized comparisons of different aspirin doses in patients with acute coronary syndromes and stable CVD (15, 24). Our study further contributed evidence supporting the efficacy of 50 mg aspirin in the older Chinese population, in either the primary or secondary prevention cohorts, which was consistent with previous findings (20). However, considering the insufficient median follow-up duration, this estimate requires a longer follow-up period.

Moreover, we examined the risks of bleeding and adverse gastrointestinal events associated with aspirin use. Aspirin is associated with an increased risk of bleeding and gastrointestinal lesions, such as mucosal erosions and ulcers (25); the gastrointestinal toxicity of aspirin is primarily attributed to the inhibition of COX-1 and COX-2 in the gastrointestinal mucosa, which impairs the physiological role of prostanoids in mucosal cytoprotection and tissue repair. Epidemiological studies and meta-analyses revealed that low-dose aspirin (≤100 mg/day) significantly increased the risk of major bleeding in East Asians compared with that in Caucasians (12, 26), especially in older individuals with complex medical conditions and multiple comorbidities, which is the focus of clinical attention. We found that aspirin 50 mg/day significantly decreased the incidence of bleeding and gastrointestinal adverse events compared with aspirin 100 mg/day, which suggests that older Chinese individuals and those at a high risk of bleeding or gastrointestinal events may benefit from aspirin 50 mg/day, in both primary and secondary prevention. In multivariable Cox regression analysis, we found that aspirin dose (100 vs. 50 mg/day), history of dyslipidemia and hemorrhage, and concomitant use of other antiplatelet and anticoagulant drugs were independent risk factors for bleeding events. Elevated levels of circulating lipoproteins have been shown to increase the sensitivity of platelets to aggregation agonists, resulting in an increased tendency toward platelet activation and thrombus formation (27). *In vitro* studies have suggested that statins enhance the antiplatelet effects of aspirin; other studies have suggested that statins could have a direct antiplatelet effect, which constitutes part of their “pleiotropic” effects, which may explain the correlation between dyslipidemia and hemorrhage related to aspirin. However, the underlying biological mechanisms are not yet completely understood. Although currently available results may assist physicians in making individual clinical judgments regarding long-term aspirin use, they are insufficient to guide routine aspirin use in the general older population. Further long-term follow-up is needed to obtain reliable evidence.

### 4.1 Limitations

We acknowledge some limitations in our study. First, this was an interim analysis of the LAPIS with a relatively short follow-up period, and selection bias could not be excluded. Regarding the endpoint analysis, approximately 20% of the cohort completed the 24-month follow-up period (median follow-up of 183 days). Notably, half of the individuals were in the initial follow-up period, which might diminish the long-term effect estimates (28, 29); the limited follow-up time was inadequate to observe the bleeding and cardiovascular events of interest. Therefore, the findings of the interim analysis support the continuation of long-term follow-up studies to determine the effects and risks of aspirin. Second, most bleeding events were minor, and only 6 (0.09%) and 1 (0.01%) of 7,021 patients had major and fatal bleeding events during follow-up, respectively, precluding the assessment of associations between severe clinical hemorrhage and aspirin dose. Third, we used PSM to control for baseline differences between the aspirin 50 and 100 mg/day groups, which may have resulted in the loss of some samples and endpoint events. Even after adjusting for potential confounders, controlling for all factors influencing the outcomes remains challenging. Fourth, we did not assess the effects on outcomes between long-term aspirin users and aspirin-naïve individuals, as the cardiovascular benefits of aspirin may continue to provide additional clinical advantages beyond the current median duration. Fifth, it was inconvenient for some patients to visit the hospital for face-to-face interviews because of coronavirus disease 2019, and follow-up via phone or WeChat may have influenced the reporting of endpoint events. Additional data from long-term follow-up of the LAPIS are required to validate our findings. Furthermore, although PCI history was recorded, the precise timing of PCI (recent vs. remote PCI) was not collected, limiting our ability to assess potential differences related to recent PCI. Finally, information regarding the specific dosages of concomitant antiplatelet and anticoagulant agents was not available in our database. Consequently, we were unable to evaluate whether the doses of these agents differed between the groups or influenced the outcomes.

Conclusively, among older adults diagnosed with CVD or cardiovascular risk factors, there was no significant difference in the risk of major cardiovascular events between daily doses of aspirin (50 or 100 mg). Furthermore, aspirin 50 mg/day was associated with a lower risk of bleeding and gastrointestinal events than aspirin 100 mg/day. The estimated effect of optimal aspirin dosage for primary and secondary prevention requires long-term follow-up data.

## Data Availability

The raw data supporting the conclusions of this article will be made available by the authors, without undue reservation.
